# Unsaturated Oral Fat Load Test Improves Glycemia, Insulinemia and Oxidative Stress Status in Nondiabetic Subjects with Abdominal Obesity

**DOI:** 10.1371/journal.pone.0161400

**Published:** 2016-08-18

**Authors:** Sergio Martinez-Hervas, Inmaculada Navarro, Jose T. Real, Ana Artero, Marta Peiro, Herminia Gonzalez-Navarro, Rafael Carmena, Juan F. Ascaso

**Affiliations:** 1 Service of Endocrinology and Nutrition, Hospital Clínico Universitario Valencia, Valencia, Spain; 2 Department of Medicine, University of Valencia, Valencia, Spain; 3 CIBER of Diabetes and Associated Metabolic Diseases (CIBERDEM), ISCIII, Madrid, Spain; 4 Hospital Clínico Research Foundation (INCLIVA), Valencia, Spain; University of Texas Health Science Center at San Antonio, UNITED STATES

## Abstract

**Aims:**

To evaluate the changes in glycemia, insulinemia, and oxidative stress markers during an oral fat load test in nondiabetic subjects with abdominal obesity and to analyze the association between postprandial oxidative stress markers and postprandial glucose and insulin responses.

**Methods:**

We included 20 subjects with abdominal obesity (waist circumference > 102 cm for men and > 88 cm for women) and 20 healthy lean controls (waist circumference < 102 cm for men and < 88 cm for women). After 12 hours of fasting we performed a standardized fat load test (0–8 hours) with supracal^®^ (50 g/m^2^). We determined metabolic parameters, oxidized and reduced glutathione, and malondialdehyde.

**Results:**

In both groups, insulin, HOMA, oxidized/reduced glutathione ratio, and malondialdehyde significantly decreased in the postprandial state after the OFLT. All these parameters were significantly higher in the abdominal obesity group at baseline and during all the postprandial points, but the reduction from the baseline levels was significantly higher in the abdominal obesity group.

**Conclusion:**

Unsaturated fat improves insulin resistance and oxidative stress status. It is possible that a consumption of unsaturated fat could be beneficial even in subjects with abdominal obesity in postprandial state.

## Introduction

Obesity and overweight are increasing health problems worldwide. It is estimated that more than 60% of all adults in the United States are overweight or obese [1]. Both conditions are closely associated with several metabolic complications [2]. The excess of body fat and particularly the visceral deposits of fat, classically known as abdominal obesity, are associated with insulin resistance (IR), impaired glucose metabolism and type 2 diabetes mellitus, as well as atherogenic dyslipidemia [3]. Moreover, obesity, in humans and in experimental models, is associated with enhanced fasting and postprandial oxidative stress [4]. Oxidative stress is also observed in conditions affecting the cardiovascular system like smoking, dyslipemia, diabetes and hypertension [5]. It is considered that oxidative stress may lead to many cellular events, such as inactivation of nitric oxidase, oxidative modifications of DNA and proteins, lipid oxidation, enhanced mitogenicity and apoptosis of cells that contribute to the development and progression of IR [6].

In the last decade, dietary habits have changed and the number of meals has increased. Thus the majority of individuals spend most of the day in a postprandial state. Postprandial lipemia (PL) is determined by the accumulation in plasma of triglyceride-rich lipoproteins (TRL) such as chylomicrons (QM), and very low density lipoproteins (VLDL) between 6–10 hours following a meal [7]. In subjects with abdominal obesity, diabetes and metabolic syndrome, the postprandial impaired clearance of lipoproteins appears to be exaggerated and this situation is related to the grade of IR [8].

Postprandial oxidative stress status is also altered in obese patients [4]. In addition, in the postprandial state, circulating lipids and lipoproteins can modulate oxidative stress status. Several fat meal test, dietary intervention and oral fat load tests (OFLT) on healthy population have shown that the type of fat can regulate oxidative stress status, such as modulation of the oxidative/antioxidative status and improve of endothelial function [9]. Beneficial effects have been shown when unsaturated fat was used compared to saturated fat [10,11]. However, scarce evidence exists about the effect of different quality of fats on oxidative stress in subjects with abdominal obesity [12].

In summary, subjects with abdominal obesity present fasting IR, altered PL and fasting and postprandial oxidative stress. Dietary saturated fat can increase fasting IR and altered PL in such subjects. Postprandial altered oxidative stress in abdominal obesity subjects can be related to dietary fat intake. However, the postprandial response of glycemia, insulinemia and its relation to postprandial oxidative stress markers in abdominal obesity subjects is not well known. Thus the aims of our study were to evaluate the changes in glycemia, insulinemia and oxidative stress markers during an OFLT in nondiabetic subjects with abdominal obesity and to analyze the association between postprandial oxidative stress markers and postprandial glucose and insulin responses.

## Subjects and Methods

### Subjects

We have studied 20 healthy volunteer subjects (11 males/9 females) and 20 subjects with abdominal obesity (7 males/13 females). All the subjects included in the study were non-hypertensive, non-diabetic, non-smokers, and had no clinical manifestations of cardiovascular disease and were off-treatment. Fasting plasma glucose was <100 mg/dl and HbA1c was < 5.7%. All the subjects included in the study had a previous OGTT. In all of the cases glucose was <140 mg/dl (Table 1). Their BMI was below 35 kg/m^2^ and their age range was between 18 and 65 years.

**Table 1:** General characteristics, fasting lipids and lipoproteins, glucose, insulin and HOMA index values in the studied groups.

	Control group (n = 20)	Abdominal obesity group (n = 20)
**Gender (M/F)**	11/9	7/13
**Age (years)**	38.8±10.1	41.0±11.9
**BMI (kg/m**^**2**^**)**	24.4±2.8	32.4±4.12 ^a^
**Waist circumference (cm)**	86.7±8.9	105.7±11.2 ^a^
**TC (mg/dL)**	178.3±27.9	213.0±24.9 ^a^
**TG (mg/dL)**	68.6±21.4	132.8±64.6 ^a^
**HDL-C (mg/dL)**	60.3±11.3	55.0±10.4
**ApoB (mg/dL)**	79.2±17.6	97.5±13.1 ^a^
**Glucose (mg/dL)**	91.5±8.3	92.8±8.6
**Insulin (μU/mL)**	5.2±2.7	19.7±14.2 ^a^
**HOMA index**	1.1±0.6	4.6±3.9 ^a^
**OGTT: Glucose 0 min (mg/dL)**	88.6±4.4	88.8±4.9
**OGTT: Glucose 120 min (mg/dL)**	87.2±12.4	111.5±13.2 ^a^

^a^ p control vs abdominal obesity group (p<0.01).

Abbreviations: BMI = body mass index;F = female; HDL-C = high density lipoprotein cholesterol; M = male; OGTT: oral glucose tolerance test; TC = total cholesterol; TG = triglycerides.

OGTT was performed before the inclusion of the subjects in the present study, in a different day.

The inclusion criteria for subjects with abdominal obesity were waist circumference >102/88 cm for men and women respectively. The inclusion criteria for subjects included in the control group were: waist circumference <102/88 cm for men and women respectively, total cholesterol (TC) concentration <200mg/dl, triglycerides (TG)<150 mg/dl and apo B <100 mg/dl, with no family history of dyslipidemia, cardiovascular disease or diabetes.

Exclusion criteria were clinical manifestations of cardiovascular disease, diabetes, hypertension, smoking habit or smoker in the previous year, consumption of > 30 g alcohol/day, intense physical fitness or weight-loss programs, body-weight fluctuation > 10% in the previous 3 months, other chronic diseases, other secondary hyperlipidemias, renal or hepatic insufficiency or hypothyroidism, use of drugs capable of modifying the lipid profile, oxidative stress or inflammation that could not be withdrawn 6 weeks before initiating the study; and any infection or inflammatory disease in the 6 weeks prior to the study.

The study was approved by the Ethical Committee of Hospital Clinico Universitario de Valencia. All the subjects gave written informed consent to participate in the study.

### Clinical and anthropometric parameters

In the study protocol the following clinical parameters were recorded: smoking, consumption of alcohol (grams of alcohol per day), physical exercise (hours/week) and use of regular or occasional drugs that could interfere with the study.

The anthropometric parameters and blood pressure were collected using standardized procedures: weight (kg), height (m), BMI (kg/m^2^), blood pressure (mmHg) and the waist circumference (midpoint between the edge lower rib and iliac crest, in centimetres). All these measurements were done by the same researcher.

### Oral fat load test

Subjects ingested a commercial liquid preparation of high-fat meal of long chain triglycerides (Supracal; SHS International Ltd; 50 g fat per m^2^ of body surface). Each 100 ml contains 50 g of fat (450 Kcal): 9.6 g are saturated, 28.2 g are monounsaturated and 10 g are polyunsaturated. The ratio ω6/ω3 is > 20/1.

The study started at 8:30 AM, after a 12 hour overnight fast. Subjects rested for 30 minutes before the first blood sample extraction. After that, the liquid preparation of lipids (Supracal) was administered. The participants remained sitting or supine during 8 hours and were only allowed to drink mineral water. Peripheral blood samples were obtained before (time 0) and at regular time intervals of 2 hours up to 8 hours after the OFLT.

### Laboratory methods

#### Measurement of lipids and lipoproteins

After 12 hours fast, blood samples were drawn from an antecubital vein in tubes containing EDTA (Vacutainer) and were centrifuged within 4 hours. Plasma was stored at 4°C for a maximum of 3 days. Total cholesterol (TC) and triglycerides (TG) levels were measured by standard enzymatic techniques. High density lipoprotein cholesterol (HDL-C) was measured after precipitation of apoB-containing lipoproteins with polyanions and VLDL cholesterol after separation of VLDL (d <1.006 g/mL) by ultracentrifugation. The LDL-C was calculated by subtraction of VLDL and HDL cholesterol from total cholesterol. Total plasma apoB was measured by immunoturbimetry. The coefficients of variation for lipids and lipoproteins were < 5%. Glucose was determined using enzymatic methods. Insulin and c-peptide values were determined using a standardized ELISA. The homeostasis model assessmentindex (HOMA), which is defined as fasting insulin (in microunits per milliliter) × fasting plasma glucose (in millimoles per liter)/22.5was used as index of insulin resistance [13]. All procedures were standard as previously described [14].

#### Oxidative stress assays

Markers of oxidative stress were determined in circulating mononuclear cells isolated by Ficoll-Hypaque methods. Oxidized and reduced glutathione (GSSG and GSH) and MDA were analysed by high-performance liquid columns (HPLC) and UV detection [15,16].

### Statistical analysis

Data were analysed using the Statistical Package for the Social Sciences (SPSS 12.1.3 for Windows; SPSS Chicago, IL, USA). For each variable, values are given as mean ± SD. Sample size was determined for a desired p value of 0.05 and 80% power to detect a postprandial difference of more than 30% in oxidative stress variables between control and subjects with abdominal obesity. A sample size of a minimum of 10 per group, matched by age, was considered satisfactory because postprandial situation should duplicate the differences expected in the fasting state.

Due to the sample size and the measurement of variables that do not fulfill the criteria of normality, non-parametric tests were used. The Mann—Whitney test was used to assess differences in measured parameters at various time intervals after the OFLT between both groups. The Wilcoxon test was used for comparison of data before and after the OFLT in the same subjects. For the comparison of proportions, Fisher’s exact test was used. The degree of relationship between two quantitative variables was analysed by the Spearman correlation coefficient.

The area under the curve (AUC) was calculated by the trapezoidal rule GraphPad Prism, version 3.0 (GraphPad Software, Inc, San Diego, Calif). Incremental integrated AUC (dAUC) was also calculated after correction for baseline values.

## Results

Clinical data and results of biochemical parameters in the fasting state are shown in Table 1. There were no significant differences in age, gender distribution, and fasting glucose values. As expected, fasting insulinemia concentrations and HOMA index were significantly higher in the abdominal obesity group. Fasting GSSG value (0.45± 0.08 vs 0.69± 0.1 U/mg prot, p<0.05), GSSH/GSH ratio (0.25± 0.05 vs 0.6 ±0.19%, p<0.01) and MDA level (0.37± 0.06 vs 0.53± 0.12 U/mg prot, p<0.05) were significantly higher in the abdominal obesity group compared to controls (Table 2). These results indicate a higher fasting oxidative stress status in the abdominal obesity group.

**Table 2:** Changes in glycemia, HOMA, insulinemia, triglyceridemia and oxidative stress markers during the oral fat load test in the studied groups.

		Fasting	2h	4h	6h	8h
**Glucose (mg/dL)**	**Control**	91.5±8.3	87.1±7.1^a^	86.1±7.8^a^	84.3±5.4^a^	84.1±6.6^a^
	**AO**	92.8±8.6^c^	92.3±6.7^c^	90.5± 8.5^c^	87.4±7.0^b^	86.7± 5.2^b^
**Insulin (μU/mL)**	**Control**	5.2±2.7	5.1±2.6	4.4±2.48	3.5±2.8^a^	3.1±1.97^a^
	**AO**	19.7±14.2^c^	15.9±7.9 8^c^	13.5±9.38^b^^,^^c^	9.5±7.28^b^^,^^c^	8.2±6.1^b^^,^^c^
**HOMA index**	**Control**	1.1±0.6	1.3±0.7	0.9±0.7	0.7±0.7	0.7±0.6
	**AO**	4.6±3.9^c^	3.7±1.9^c^	3.2±2.3^c^	2.1±1.8^b^^,^^c^	1.7±1.3^b^^,^^c^
**C peptide (ng/mL)**	**Control**	1.8±0.9		1.6±0.9^a^		1.3±0.7^a^
	**AO**	2.8±1.3^c^		2.5±1.2^c^		2.0±0.9^b^^,^^c^
**Triglycerides(mg/dl)**	**Control**	68.6±21.4	107.9±49.1^a^	122.0±59.4^a^	95.1±66.4^a^	65.6±20.4^a^
	**AO**	132.8±64.6^c^	178.3±67.9^b^^,^^c^	216.0±73.6^b^^,^^c^	148.4±49.0^b^^,^^c^	111.5±36.4^b^^,^^c^
**GSSG (U/mg prot)**	**Control**	0.45±0.08	0.36±0.07^a^	0.31±0.07^a^	0.28±0.05^a^	0.23±0.05^a^
	**AO**	0.69±0.1^c^	0.43±0.09^b^^,^^c^	0.38±0.08^b^^,^^c^	0.33±0.82^b^^,^^c^	0.26±0.84^b^
**GSH (U/mg prot)**	**Control**	20.1±2.3	22.3±1.8^a^	23.3±2.0^a^	24.3±1.7^a^	25.4±1.7^a^
	**AO**	10.8±3.1	12.5±2.8^b^	14.1±3.0^b^	15.7±3.8^b^	17.1±3.6^b^
**GSSG/GSH x10^1^**	**Control**	0.25±0.05	0.16±0.03^a^	0.13±0.03^a^	0.11±0.02^a^	0.09±0.02^a^
	**AO**	0.6±0.19^c^	0.35±0.07^b^^,^^c^	0.27±0.06^b^^,^^c^	0.22±0.49^b^^,^^c^	0.15±0.41^b^^,^^c^
**MDA (U/mg prot)**	**Control**	0.37±0.06	0.34±0.03^a^	0.30±0.06^a^	0.22±0.06^a^	0.22±0.06^a^
	**AO**	0.53±0.12^c^	0.34±0.04^b^	0.24±0.08^b^^,^^c^	0.19±0.04^b^^,^^c^	0.18±0.02^b^^,^^c^

^a^ p< 0,05 fasting vs postprandial in control group.

^b^ p< 0,01 fasting vs postprandial in AO group.

^c^ p< 0.01 control vs AO group.

Abbreviations: AO = abdominal obesity;GSH: reduced glutathione;GSSG = oxidizedglutathione; MDA: malonildyaldehide.

C-peptide was analyzed at time 0, 4 and 8 hours.

**Fig 1:**
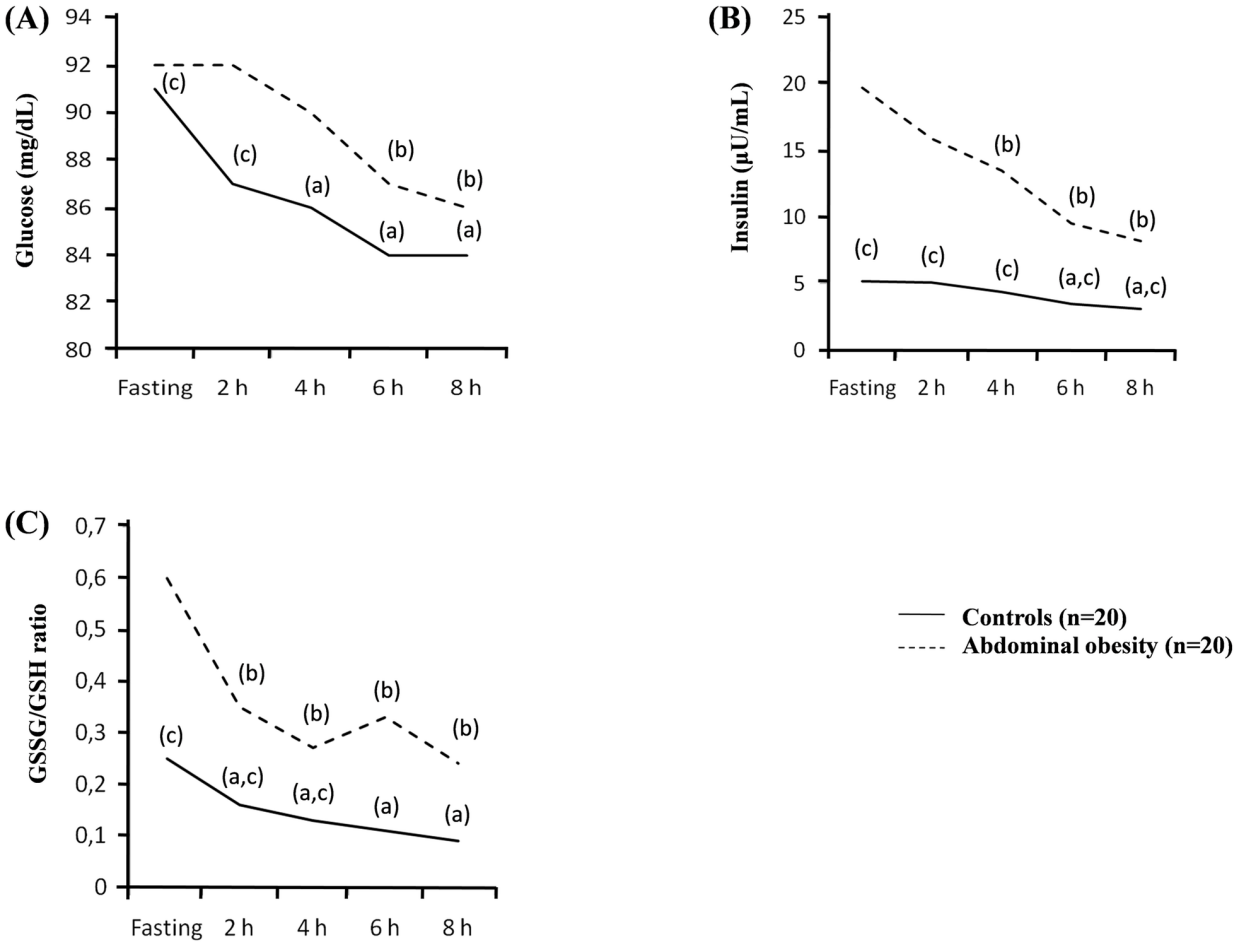
Plasmatic changes during the oral fat load test in control subjects and subjects with abdominal fat deposits. (A) Glycemia during the oral fat load test. (B) Insulinemia during the oral fat load test. (C) GSSG/GSH ratio during the oral fat load test. (a) p < 0.05 fasting vs postprandial in control group. (b) p < 0.01 fasting vs postprandial in Abdominal obesity group. (c) p <0.01 control vs Abdominal obesity group.

Postprandial values of the analyzed parameters are shown in Table 2. During the OFLT we found a significant increase in postprandial triglycerides plasma values achieving the maximum at 4 hours and recovering basal values after 8 hours. However, a significant decrease in glycemia, insulinemia, and c-peptide levels was observed in both groups (Table 2, Fig 1). The decrease of insulinemia compared to fasting values was significantly higher in the abdominal obesity group compared to the control group. In abdominal obesity group insulinemia at 6 h decreased from 19.7 to 9.5 (50%) and at 8 h to 8.2 (60%). C-peptide levels decreased progressively compared to fasting values, showing significant differences at 4 and 8 h in controls, and at 8 h in subjects with abdominal obesity. We also found that after the OFLT there was a significant decrease in GSSG/GSH ratio at 2, 4, 6 and 8 hours compared to fasting levels in both groups. The MDA value, a byproduct of lipid peroxidation and marker of oxidative stress, also significantly decreased after the OFLT in both groups (Table 2, Fig 1).

In Table 3 are shown the AUC and dAUC. We found significant differences between control and abdominal obesity group.

**Table 3:** AUC and dAUCglycemia, insulinemia, triglyceridemia and oxidative stress markers during the oral fat load test.

	Control group (n = 20)	Abdominal obesity group (n = 20)
**AUC glucose(mg x dL**^**-1**^ **x h**^**-1**^**)**	690.6±49.5	720.0±50.57 ^a^
**dAUC glucose(mg x dL**^**-1**^ **x h**^**-1**^**)**	45.3±36.6	44.7±42.3
**AUC insulin(μU x mL**^**-1**^ **x h**^**-1**^**)**	34.4±16.4	105.7±61.6 ^a^
**dAUCinsulin(μU x mL**^**-1**^ **x h**^**-1**^**)**	15.2±13.2	58.6±64.2 ^a^
**AUC HOMA(h**^**-1**^**)**	10.5±14.9	21.6±10.6 ^a^
**dAUC HOMA (h**^**-1**^**)**	3.7±3.3	14.3±19.8 ^a^
**AUC TG(mg x dL**^**-1**^ **x h**^**-1**^**)**	784.0±330.5	1329.5±438.4 ^a^
**dAUC TG(mg x dL**^**-1**^ **x h**^**-1**^**)**	258.7±192.1	338.9±176.8
**AUC GSSG(U x mg prot**^**-1**^ **x h**^**-1**^**)**	2.6±0.5	3.3±0.5 ^a^
**dAUC GSSG(U x mg prot**^**-1**^ **x h**^**-1**^**)**	0.96±0.42	2.3±0.6 ^a^
**AUC GSH(U x mg prot**^**-1**^ **x h**^**-1**^**)**	185.1±12.5	112.8±23.4 ^a^
**dAUC GSH(U x mg prot**^**-1**^ **x h**^**-1**^**)**	23.9±18.6	25.9±16.1
**AUC GSSG/GSH (h**^**-1**^**)**	0.12±0.02	0.25±0.5 ^a^
**dAUC GSSG/GSH (h**^**-1**^**)**	0.06±0.03	0.29±0.12 ^a^
**AUC MDA(U x mg prot**^**-1**^ **x h**^**-1**^**)**	2.4±0.4	2.3±0.3
**dAUC MDA(U x mg prot**^**-1**^ **x h**^**-1**^**)**	0.69±0.2	1.9±0.8 ^a^

^a^ p control vs abdominal obesity group (p<0.01).

Abbreviations: GSH: reduced glutathione; GSSG = oxidized glutathione; MDA: malonildyaldehide.

Finally, we also evaluated the correlations between two variables (Table 4). Markers of oxidative stress were significantly associated with HOMA, TG, the peak of TG (4 hours) and dAUC of HOMA. The dUAC of TG did not show significant association. Furthermore dAUC of HOMA was correlated with dAUC of GSSG/GSH ratio. However no correlation was found with dAUC of TG.

**Table 4:** Correlation coefficients among oxidative stress markers and markers of insulinresistance.

	GSSG/GSH	dAUC GSSG/GSH (h^-1^)	dAUC HOMA (h^-1^)
**HOMA index**	r 0.659 p<0.001	r 0.646 p<0.001	r 0.756 p<0.001
**TG (mg/dL)**	r 0.608 p<0.001	r 0.610 p<0.001	r 0.611 p<0.001
**Peak of TG (mg/dL; 4 h)**	r 0.454 p 0.003	r 0.423 p 0.006	r 0.537 p<0.001
**GSSG (U/mg prot)**	r 0.858 p<0.001	r 0.819 p<0.001	r 0.442 p 0.004
**GSH (U/mg prot)**	r -0.924 p<0.001	r -0.929 p<0.001	r -0.444 p 0.004
**GSSG/GSH**		r 0.977 p<0.001	r 0.471 p0.002
**MDA (U/mg prot)**	r 0.577 p<0.001	r 0.492 p<0.001	r 0.287 p0.073
**dAUC glucose (mg x dL**^**-1**^ **x h**^**-1**^**)**	r 0.011 p0.946	r 0.008 p0.961	r 0.014 p0.932
**dAUC insulin (μU x mL**^**-1**^ **x h**^**-1**^**)**	r 0.557 p<0.001	r 0.567 p<0.001	r 0.871 p<0.001
**dAUC HOMA (h**^**-1**^**)**	r 0.471 p0.002	r 0.470 p0.002	
**dAUC TG (mg x dL**^**-1**^ **x h**^**-1**^**)**	r 0.134 p0.409	r 0.093 p0.570	r 0.292 p0.068
**dAUC GSSG/GSH (h**^**-1**^**)**	r 0.977 p<0.001		r 0.470 p0.002

Abbreviations:GSH: reduced glutathione; GSSG = oxidized glutathione; MDA: malonildyaldehide; TG = triglycerides.

## Discussion

In our study, non diabetic subjects with abdominal obesity had fasting HOMA index values fourth fold higher than controls, higher fasting triglyceridemia and higher fasting oxidative stress markers. Moreover, significantly higher postprandial triglyceridemia was observed after an OLFT. As expected, these results indicate that abdominal obesity can be considered as a model of fasting IR and altered PL.

We have found that during the OFLT, glucose and insulin values significantly decreased in abdominal obesity and control groups with a higher decrease in abdominal obesity subjects (50% at 6 h and 60% at 8 h in the abdominal obesity group). We also found that c-peptide levels decreased in both groups (approximately 10% at 4 h and 30% at 8 h in both groups). Previous studies have shown that the intake of sugars, especially fructose, has been associated with IR in rats [17]. In contrast to our results, hyperlipidemia generated by intravenous infusion of lipids consistently induces acute insulin resistance. However, when hyperlipidemia is eliminated, glucose tolerance returns to the normal range [18].

Many mechanisms have been implicated in the induction of insulin resistance in the postprandial state. In human models of insulin resistance such as subjects with abdominal obesity, metabolic syndrome, obesity and type 2 diabetes, the increased oxidation of energetic substrates generated by excessive postprandial TG or FFA can activate various serine kinases that negatively affect insulin action inducing IR [19,20]. Furthermore, an excess of postprandial FFA disposal induces a metabolic shift toward increased reliance on fat relative to glucose for energy production. Experimentally, this shift increases mitochondrial H2O2 production as has been shown in skeletal muscle, adipose tissue, and kidney of mice fed with a high-fat diet [21–23]. In contrast, the suppression of beta-oxidation or selective scavenging of mitochondrial H2O2 preserves skeletal muscle insulin sensitivity in models of diet-induced obesity [20,24].

In addition, insulin resistance states show fasting and postprandial increase of oxidative stress markers. In fact, oxidative stress has been implicated in the development of insulin resistance [25]. We have found that fasting oxidative stress markers are significantly increased in abdominal obesity subjects compared to controls and are significantly related to anthropometric and biochemical markers of IR. Mechanisms by which high dietary fat and obesity induce overproduction of ROS include infiltration of macrophages into adipose tissue, release of proinflammatory adipokines, elevated expression of NADPH oxidase (Nox), and enhanced generation of oxidative stress by mitochondria [26,27]. In this line, increased levels of lipid peroxidation products and markers of oxidized DNA have been found in plasma, skeletal muscle and urine from obese individuals [28]. More recently, elevated levels of oxidized proteins have been observed in subcutaneous adipose tissue [29,30].

In contrast to previous discussion, our study has demonstrated a significant reduction of postprandial glycemia, insulinemia, c-peptide and oxidative stress markers using an acute oral overload of unsaturated fat. We have found a significant correlation between oxidative stress markers and postprandial lipemia. There is an increase of TG achieving the maximum peak four hours after the beginning of the test. However, although postprandial lipemia has been implicated in the development of insulin resistance and oxidative stress, and despite the increase of TG, there are significant reductions of the HOMA index and oxidative stress markers.

The influence of dietary macronutrients in insulin sensitivity is not well known. Previous evidence suggests that dietary fat might also impact on insulin sensitivity in humans. Experimental and interventional studies using saturated fat have demonstrated an increase in fasting and postprandial IR [31–34]. In addition, a high membrane content of phospholipids rich in saturated fat can alter phyco-chemical properties decreasing the recruitment of GLUT. In contrast, a more fluid membrane with unsaturated fat can improve insulin sensitivity [35]. Iggman et al demonstrated in elderly men that palmitic acid, the major saturated fatty acid found in adipose tissue, inversely correlates to insulin sensitivity measured by euglucemic-hyperinsulinemic clamp. However, they also found a positive relation of insulin sensitivity with the content of linoleic acid in adipose tissue [36]. It is in accordance with our results because our commercial liquid preparation of high-fat meal of long chain triglycerides is composed in the majority by linoleic acid (59%). Furthermore, in line with our findings, the replacements of dietary saturated fat by unsaturated fat also improved fasting insulin sensitivity [37]. Several other studies have demonstrated that unsaturated fat improves fasting and postprandial IR, although the mechanism is largely unknown [38]. Moreover the PREDIMED study has recently demonstrated that unsaturated fat can improve fasting insulin sensitivity and prevent the incidence of type 2 diabetes [39].

In the same line, the type of fat can also regulate oxidative stress status [9]. Unsaturated fat has shown beneficial effects [10,11]. We have found a significant reduction in oxidative stress markers throughout the fat load test. Finally, a postprandial reduction in oxidative stress markers can improve insulin sensitivity, as observed in our study and others [24,40,41]. In fact, the decrease of HOMA significantly correlated to postprandial decreases of oxidative stress markers and insulin, but not glucose. Furthermore dAUC GSSG/GSH only correlated significantly with dAUC HOMA and dAUC insulin. There was no significant association with dAUC glucose. It is in accordance with the findings of Delarue et al who found that after a dietary substitution of dietary fat by unsaturated fat, oral glucose load induced a significant decrease in insulinemic response without alteration of the glucose response [42].

Our study has some limitations. The OFLT is an acute intervention thus we cannot extrapolate long term beneficial effects of unsaturated fat on postprandial glycemia, insulinemia and oxidative stress status. In the same line, the use of a test with commercial liquid preparation of fat is a non physiological ingestion of fat. Furthermore, since OFLT was not associated with an oral glucose load it is not possible assess in depth insulin resistance and stimulated insulin secretion after oral fat load. We have not measured the metabolic parameters (glucose, insulin and oxidative stress markers) some hours later of the OFLT to assess that the reduction in this parameters was dependent of the prolonged fasting state. Finally, oxidative stress markers analyzed could be also altered by others players regulating the postprandial state.

In summary, after an OFLT using predominantly unsaturated fat, glycemia, insulinemia and oxidative stress markers significantly decreased in healthy lean subjects and nondiabetic abdominal obesity subjects. The exact mechanism to explain these results is unknown. We speculate that the use of unsaturated fat and the significant decrease of postprandial oxidative stress markers observed can be responsible, among several other mechanisms, of the postprandial glycemia and insulinemia decrease. However, more studies are necessary to confirm the postprandial beneficial effect on glucose and insulin plasma values observed using unsaturated fat.

## Supporting Information

S1 File Data used for the analysis. (SAV)
